# Low Input Whole-Exome Sequencing to Determine the Representation of the Tumor Exome in Circulating DNA of Non-Small Cell Lung Cancer Patients

**DOI:** 10.1371/journal.pone.0161012

**Published:** 2016-08-16

**Authors:** Steffen Dietz, Uwe Schirmer, Clémentine Mercé, Nikolas von Bubnoff, Edgar Dahl, Michael Meister, Thomas Muley, Michael Thomas, Holger Sültmann

**Affiliations:** 1 Cancer Genome Research Group, German Cancer Research Center (DKFZ) and National Center for Tumor Diseases (NCT), Im Neuenheimer Feld 460, Heidelberg, Germany; 2 Translational Lung Research Center (TLRC), German Center for Lung Research (DZL), Heidelberg, Germany; 3 Department of Hematology, Oncology and Stem Cell Transplantation, University Medical Center Freiburg, Freiburg, Germany; 4 German Cancer Consortium (DKTK), Heidelberg, Germany; 5 Molecular Oncology Group, Institute of Pathology, RWTH Aachen University, Aachen, Germany; 6 Translational Research Unit, Thoraxklinik at University Hospital Heidelberg, Heidelberg, Germany; 7 Department of Oncology, Thoraxklinik at University Hospital Heidelberg, Heidelberg, Germany; CNR, ITALY

## Abstract

Circulating cell-free DNA (cfDNA) released from cancerous tissues has been found to harbor tumor-associated alterations and to represent the molecular composition of the tumor. Recent advances in technologies, especially in next-generation sequencing, enable the analysis of low amounts of cfDNA from body fluids. We analyzed the exomes of tumor tissue and matched serum samples to investigate the molecular representation of the tumor exome in cfDNA. To this end, we implemented a workflow for sequencing of cfDNA from low serum volumes (200 μl) and performed whole-exome sequencing (WES) of serum and matched tumor tissue samples from six non-small cell lung cancer (NSCLC) patients and two control sera. Exomes, including untranslated regions (UTRs) of cfDNA were sequenced with an average coverage of 68.5x. Enrichment efficiency, target coverage, and sequencing depth of cfDNA reads were comparable to those from matched tissues. Discovered variants were compared between serum and tissue as well as to the COSMIC database of known mutations. Although not all tissue variants could be confirmed in the matched serum, up to 57% of the tumor variants were reflected in matched cfDNA with mutations in *PIK3CA*, *ALK*, and *PTEN* as well as variants at COSMIC annotated sites in all six patients analyzed. Moreover, cfDNA revealed a mutation in *MTOR*, which was not detected in the matched tissue, potentially from an untested region of the heterogeneous primary tumor or from a distant metastatic clone. WES of cfDNA may provide additional complementary molecular information about clinically relevant mutations and the clonal heterogeneity of the tumors.

## Introduction

Since circulating cell-free DNA (cfDNA) was first shown to carry somatic aberrations, its utility for molecular characterization of tumor diseases has been demonstrated in several recent studies [1–4]. Thus, the analysis of cfDNA has become one focus of biomarker research in molecular oncology. Currently, tissue biopsies are still the gold standard for molecular genotyping of tumor diseases. However, tissue biopsies are associated with the risk of invasive procedures and often provide only limited information about the heterogeneous molecular composition of the tumor and its genetic causes. Especially characterization of spatial and temporal intra-tumor heterogeneity of primary and metastatic lesions requires unfeasible serial sampling from multiple sites, indicating the strong need for less invasive approaches [5, 6]. CfDNA, easily to obtain from blood, is a potential source of diagnostic and prognostic biomarkers. Recent studies demonstrated the analysis of cfDNA as potential minimal-invasive surrogate for cancer diagnostics and prognostics [7–9]. Sequential characterization of genetic aberrations in cfDNA has been demonstrated for dynamic therapy monitoring and as an indicator of molecularly manifested resistance [10–12]. Moreover, detection of cfDNA in the circulation of cancer patients after surgery could potentially indicate minimal residual disease, which may eventually lead to disease recurrence [13, 14].

Recent technological advances, especially in sequencing and digital PCR technologies, allow the analysis of low amounts of circulating DNA from different body fluids. To date, BEAMing and digital (droplet) PCR have been introduced to detect and track mutations in cfDNA in plasma and serum from cancer patients [8, 15, 16]. These technologies are predominantly used for the analysis of mutational hotspots, as they require previous knowledge of the mutation sites. In addition, the poor integrity of cfDNA, which is typically of about 166 bp in size [17], considerably reduces the efficiencies of all PCR-dependent approaches. In contrast, next generation sequencing allows global identification of molecular variants leading to malignant transformation at a genome-wide scale. In the past decade, large international sequencing consortia have revealed various cancer-associated somatic alterations and have led to a better understanding of the complex molecular composition of tumors, e.g. non-small cell lung cancer (NSCLC) [18, 19]. Only few prominent cancer genes were found to be recurrently mutated at high frequencies among multiple tumor types, whereas the majority of somatic events are present at lower frequencies [20–22]. In NSCLC, which is the leading cause of malignancy-related mortality [23], patient tissues often harbor activating mutations in *KRAS* or in members of the *ERBB* gene family as well as loss-of-function mutations in the tumor suppressor gene *TP53* [19]. However, comprehensive molecular genotyping efforts also revealed a broad mutational spectrum [18, 19]. Hence, since cancer harbors individual mutational signatures, exome sequencing offers the advantage to identify individual coding and UTR mutations aside from the prominent mutational hotspots. Different approaches including whole-genome as well as targeted deep sequencing of cancer-associated loci in cfDNA have been reported for cancer genotyping [1, 2, 12, 24]. Furthermore, recent proof-of-concept studies illustrate the utility of whole-exome sequencing (WES) of cfDNA for disease monitoring under therapy in several cancer entities, including NSCLC. [11, 25, 26]. Besides profiling of disease-associated genetic variants, exome sequencing further enables the identification of emerging molecular resistance markers. However, up to date there is no general consensus or standardized method for the analysis and WES of cfDNA and most commonly available technologies require large amounts of starting material. Moreover, the molecular representation of the complex tumor exome in cfDNA has not yet been investigated comprehensively.

Here, we evaluated WES to assess the exomes of six NSCLC patients in primary tumor and corresponding serum samples. To this end, we implemented a workflow for WES from low volumes of 200 μl serum by combining an ultra-low input library preparation protocol with a hybridization-based exome enrichment technology. Our results provide evidence for cfDNA to inform about the molecular constitution of the disease in the six advanced cancer patients with up to 57% of the tumor variants represented in the matched serum samples. By comparing gene sets of frequently mutated genes and the COSMIC database to WES data, we identified common cancer associated mutations (e.g. *PIK3CA*, *ALK*, *MAP2K3*, and *PTEN*) in serum and tissue pairs. Moreover, we detected additional mutations of clinical relevance in cfDNA, including a potentially actionable mutation in *MTOR*, which were not found in the primary tumors. In summary, we show that WES of cfDNA informs about the primary tumors’ molecular alterations and can provide complementary information about the mutational patterns in distant clones.

## Materials and Methods

### Sample collection

Tumor tissue and corresponding serum from six NSCLC patients was collected at the Thoraxklinik Heidelberg and provided via LungBiobiank Heidelberg. Of the six cases, three were diagnosed with lung adenocarcinoma (LUAD) and three with squamous cell carcinoma (SCC). All patients had provided written informed consent. Blood was collected in S-Monovette 7,5ml Z-Gel tubes (Sarstedt, Nürmbrecht, Germany), allowed to clot for 60 min and then centrifuged for 10 min at 2,000 × g at 10°C. Serum was stored −80°C until use. Two serum pools were collected at the Thoraxklinik Heidelberg and used as control and for protocol implementation. Tissue samples were examined for tumor cell content by pathologists, snap-frozen and stored at -80°C. The study was approved by the local ethics committee of the Medical Faculty Heidelberg (270/2001) with amendment 3 (July 31, 2014).

### Isolation and QC of circulating DNA

DNA was isolated from 200 μL serum using the QIAamp DNA Blood Mini Kit (Qiagen, Hilden, Germany). To ensure efficient lysis of DNA-bound proteins, serum was subjected to proteinase K digestion at 37°C for 1h. Purified cfDNA was quantified by digital PCR using the QuantStudio 3D System (Thermo Fischer Scientific, Waltham, MA, USA). Allele copies of the *TERT* locus in plasma DNA were quantified and the DNA amount was calculated based on an external standard reference curve of fragmented genomic DNA. Briefly, 3 μL of purified cfDNA were mixed with 7.25 μL QS3D Master Mix v2, 0.75 μL TaqMan Copy Number Reference Assay *TERT* (Thermo Fischer Scientific), and 3.5 μl water. Due to the low integrity of cfDNA, genomic DNA (Roche Diagnostics, Mannheim, Germany) of the external standard curve was sheared to the same length in order to compensate for the influence of the DNA integrity on PCR reactions and quantity estimations. The integrity of cfDNA was examined by capillary electrophoresis on a Bioanalyzer 2100 system with the High Sensitivity DNA Kit (Agilent Technologies, Santa Clara, CA, USA). Approximately 500 pg cfDNA was used for Bioanalyzer analysis. Digital PCR chips were loaded, thermal cycled, and analyzed according to the manufacturer`s instructions.

### Isolation of genomic DNA from tumor tissues

Fresh frozen tumor tissue was homogenized using a TissueLyser II (Qiagen) and genomic DNA was extracted using the AllPrep DNA/RNA/miRNA Universal Kit (Qiagen) according to the manufacturer’s protocol. DNA concentrations were determined using a Nanodrop ND-1000 spectrophotometer.

### Library preparation and exome enrichment

Prior to library preparation, tissue and serum DNA was sheared to an average fragment length of 150 bp using a S220 Focused-ultrasonicator (Covaris, Woburn, MA, USA). Sequencing libraries were prepared by adapter ligation and PCR amplification using the ThruPLEX-FD Prep Kit (Rubicon Genomics, Ann Arbor, MI, USA) according to the manufacturer’s instructions. Starting from approximately 10 ng of cfDNA, libraries were generated using a total of 11 amplification cycles, consisting of four cycles to fuse the index adapters with the prepared template molecules and seven amplification cycles. Corresponding tumor tissue libraries were prepared from 50 ng DNA using seven amplification cycles. To reduce the number of PCR duplicates in sequencing reads and to avoid amplification biases, EvaGreen was added to the PCR reaction master mix and the amplification was monitored in real time. Once the PCR reaction had reached the exponential amplification phase, it was terminated. The number of required PCR cycles was evaluated in previous experiments. Different barcodes were used for library indexing to allow sample pooling for multiplexed exome capture and sequencing. Hybridization-based exome enrichment was performed using the Agilent SureSelect^*XT2*^ All Exon v5 + UTR target enrichment system (Agilent Technologies, Santa Clara, CA, USA). Equal amounts of 215 ng of 7 multiplexed libraries (3 from serum and corresponding tissues as well as 1 from pooled control serum) were combined for enrichment. Universal Blocking Oligos (Integrated DNA Technologies, Coralville, IA, USA) were added to the library pools to ensure compatibility of the hybridization probes with ThruPLEX libraries. Captured libraries were amplified independently in two separate PCR reactions and pooled again afterwards. Library sizes and qualities were evaluated pre- and post-exome enrichment by Bioanalyzer 2100 analysis using the High Sensitivity DNA Kit (Agilent Technologies) and quantified using the Qubit dsDNA HS Assay kit (Thermo Fischer Scientific). Enriched multiplexes were subjected to 100 bp paired-end sequencing using the Illumina HiSeq 2000 v3 at the DKFZ Genomics and Proteomics Core Facility. Each 7-plexed library pool was loaded on two lanes in order to increase the read count per sample.

### NGS data processing

A custom computational analysis pipeline was implemented for WES data processing as well as comparison of variants called from tumor tissue and matched serum samples. Upon quality score estimation using FastQC (v0.11.5), FASTQ files were aligned to the human genome (hg19/ GRCh37) using BWA v0.7.4 [27]. Mapping statistics were calculated using SAMtools (v0.1.19) [28] and target enrichment quality and target coverage was assessed using the R Target Enrichment Quality Control (TEQC 3.2.0) package [29] and a custom R script (http://www.gettinggeneticsdone.com/2014/03/visualize-coverage-exome-targeted-ngs-bedtools.html). PCR duplicates were removed using Picard MarkDuplicates (Picard tools v1.129). Mapped reads were locally realigned around known insertion and deletion sites [30] and recalibrated using RealignerTargetCreator, IndelRealigner, and BaseRecalibration from GATK (v3.5–0) [31].

### Variant calling and processing

Variants and small INDELs were called using HaplotypeCaller from GATK (v3.5–0). Annotation and effect prediction of identified variants was performed using snpEff 4.1g [32]. Since no matched normal tissue or germline DNA was available from the tumor patients, all variants were subsequently filtered. Variants present in the dbSNP database (dpSNP138) were considered as SNPs and removed. Variants in tumor tissues were only retained for further analysis if they had a mutant allele frequency between 20% and 80% (above 80% was considered as homozygous and thus as germline variant), a minimum sequencing depth of 20x, and a minimum base quality of 50. Variants in cfDNA with a sequencing depth <10x were removed. Variants in tumor tissue were compared with those in the corresponding serum using VCFtools (v0.1.12b) [33]. We further excluded identical variants identified in more than 2 patients, as these are most likely technical artifacts. To identify cancer relevant mutations, variants from NSCLC tissue and serum DNA were compared to the COSMIC database of known somatic mutations. Since no matched normal tissue was available from NSCLC patients, we designed gene sets for LUAD and SCC based on the frequency of mutations listed in the COSMIC and TCGA datasets: The LUAD set of 58 genes was built based on the most frequently mutated genes in the TCGA [19] and COSMIC database for LUAD, a public NSCLC gene panel [12], and the COSMIC top 20 cancer genes for LUAD (S1 Table). The SCC set of 45 genes was designed based on the most frequently mutated genes in the TCGA [18] and COSMIC database for SCC, a published NSCLC gene panel r [12], and the COSMIC top 20 cancer genes for SCC (S2 Table). Variants in tumor tissues and corresponding cfDNA were screened for mutations in genes of the LUAD and SCC sets using VCFtools (v0.1.12b) [33] and visualized in the Integrative Genomics Viewer (IGC v.2.3) [34].

### Sanger Sequencing

Prior to Sanger sequencing, a 98 bp fragment spanning the *MTOR* mutation c.4228 C>A (p.P1410T) in patient 4 was amplified using the KAPA High Fidelity HotStart PCR kit (Kapa Biosystems, Wilmington, MA, USA). The PCR reaction contained 1X KAPA HiFi Fidelity buffer, 0.3 mM each dNTP, 0.3 μM forward primer (5´-GAGGACCGTCGCTTGGTG -3´), 0.3 μM reverse primer (5´- CGAGCATATGCCAAAGCACT—3´), 0.5 U KAPA HiFi HotStart DNA Polymerase, and 5 ng cfDNA or 20 ng tumor tissue DNA in a total volume of 25 μl per reaction. Cycling conditions were as follows: Initial denaturation at 95°C for 3 min, 35 cycles of 98°C for 20 s, 62°C for 15 s, and 72°C for 30 s, followed by a final extension at 72°C for 5 min. The PCR products were purified using the QIAquick PCR Purification Kit (Qiagen) according to the manufacturer’s protocol. Sequencing was performed at GATC Biotech AG (Konstanz, Germany).

## Results

Of the six NSCLC patients analyzed, three were female and three male. All patients were diagnosed with advanced, lymph node-positive stage III tumors, three SCC and three LUAD. All patients included had a smoking history. Patient data and clinical characteristics are summarized in (Table 1).

**Table 1:** Patient characteristics.

Patient	Gender	Smoking history (py)	Tumor type	Stage	TNM	Diameter
P1	F	former smoker (40 py)	SCC	III A	pT4 N1 M0	5.5 cm
P2	M	smoker (40 py)	SCC	III A	pT3 N2 M0	8 cm
P3	M	former smoker (50 py)	LUAD	III A	pT4 N1 M0	11.2 cm
P4	F	former smoker (15 py)	LUAD	III B	pT4 N2 M0	7.2 cm
P5	M	former smoker (-)	LUAD	III B	pT4 N2 M0	9.5 cm
P6	F	smoker (60 py)	SCC	III B	pT4 N2 M0	5.5 cm

(F: female; M: male; py: packyears; SCC: squamous cell carcinoma, LUAD: lung adenocarcinoma)

### Experimental platform and serum processing

**Fig 1:**
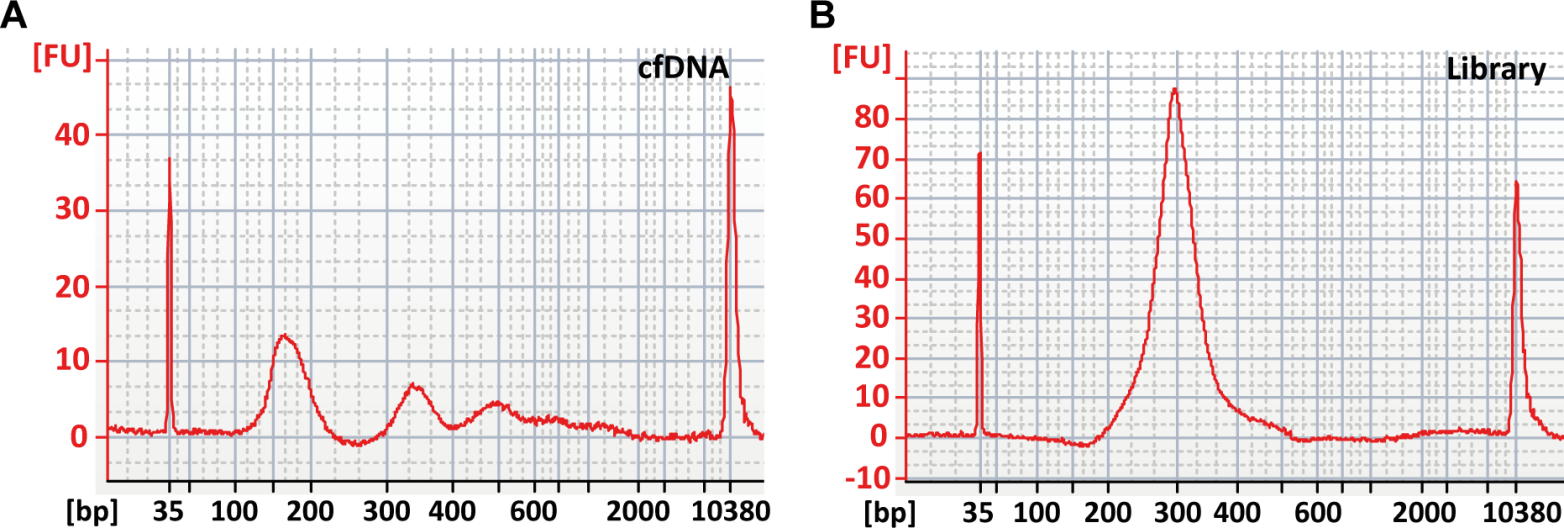
Integrity of cfDNA and a corresponding sequencing library. (A) Integrity and size distribution of cfDNA fragments from patient 1 showing a nucleosomal laddering of cfDNA with fragment sizes of 166, 360, and 515 bp; (B) Corresponding sequencing library from patient 1, prepared from 10ng cfDNA.

To investigate genomic alterations in cfDNA, we initially implemented an experimental and computational workflow (S1 Fig) for WES analysis of cfDNA from low volumes of serum and matched tumor tissue samples. Information including yields and input amounts from each step of the workflow are summarized in Table 2. Starting from 200 μL serum, purified cfDNA was quantified by digital PCR. Quantification revealed a wide range of cfDNA amounts from 131 ng/mL to 1,168 ng/mlL serum. The recovery from 200 μL was higher in sera from NSCLC patients (median: 76.01 ng; range: 26.22–233.67 ng), compared to pooled control sera (median: 34.82 ng; range 24.5–45.13 ng). To assess the integrity of cfDNA, we performed capillary gel electrophoresis. Quality assessment also revealed variance between the samples and clear differences in the integrity and size distribution of cfDNA fragments. Profiles of all serum samples revealed an accumulation of short DNA molecules with a predominant fragment size of 166 bp, which is in correspondence with the nucleosomal appearance of circulating DNA fragments bound to a nucleosome plus linker histones [35–37]. No difference was observed between serum DNA from cancer patients and control subjects. However, sizing of cfDNA from NSCLC patients further revealed a di- and trinucleosomal fragmentation pattern with molecules of multiples of this size (Fig 1A). We observed cfDNA with a median fragment length of about 360 and 541 bp in four of the six cases (data not shown), representing a (oligo-) nucleosomal laddering and thus indicating the potential origin of cfDNA from cellular DNA cleavage during apoptosis [38, 39]. Previous reports have shown a correlation between the biphasic pattern of plasma DNA fragments and the number of circulating tumor cells (CTCs) as well as elevated plasma DNA concentrations [40]. Further, an increased percentage of mutated DNA molecules in the circulation of cancer patients with biphasic plasma DNA size distribution was noted [40]. In addition, we detected high molecular weight DNA in the sera of patients 2 and 4.

**Table 2:** Sample characteristics and quality metrics of the sequencing data from cfDNA and corresponding tumor tissues.

Patient	Sample	ctrl1	ctrl2	P1	P2	P3	P4	P5	P6	median (P1-P6)
**DNA amount (ng/mL serum)**	**Serum**	123	226	314	1168	620	446	298	131	**380**
**Fragment size**	**Serum**	173	169	166	178	166	165	177	159	**166**
**Library insert size**	**Serum**	148	149	168	175	163	165	164	167	**166**
	**Tissue**	**-**	**-**	145	134	127	128	132	141	**133**
**GC content (%)**	**Serum**	47	45	47	48	48	47	47	47	**47**
	**Tissue**	**-**	**-**	48	46	45	44	46	45	**45.5**
**Number of raw reads (mio.)**	**Serum**	166	118	140	182	136	181	190	107	**160.5**
	**Tissue**	**-**	**-**	192	91	161	145	159	119	**152**
**Propely paired reads (mio.)**	**Serum**	139	100	120	157	115	155	162	91	**137.5**
	**Tissue**	**-**	**-**	160	78	140	126	126	103	**126**
**Median target coverage**	**Serum**	80x	49x	63x	74x	48x	85x	77x	38x	**68.5x**
	**Tissue**	**-**	**-**	92x	39x	71x	57x	65x	54x	**61x**
**Targets with coverage >20x (%)**	**Serum**	66	62	64	64	60	66	64	58	**64**
	**Tissue**	**-**	**-**	67	61	65	63	65	63	**64**
**High quality filtered reads (mio.)**	**Serum**	26.8	15.96	17	14.56	11.44	23.31	16.3	11.15	**15.43**
	**Tissue**	**-**	**-**	39.59	36.15	36.93	39.86	54.84	38.66	**39.125**
**Number of variants called**	**Serum**	53,728	43,232	43,315	37,170	32,350	46,716	39,933	34,273	**38,552**
	**Tissue**	**-**	**-**	50,084	44,876	49,024	47,245	49,105	48,080	**48,552**
**Number of variants not in dbSNP**	**Serum**	11,449	8,966	9,733	8,305	7,255	10,782	9,090	7,299	**8,698**
	**Tissue**	**-**	**-**	12,678	10,985	12,937	11,943	11,845	11,253	**11,894**
**Filtered variants**	**Serum**	7,623	5,049	2,660	1,073	589	4,105	1,759	769	**1,416**
	**Tissue**	**-**	**-**	3,322	1,892	2,861	2,232	2,820	2,294	**2,557**
**Common variants in serum + tissue**	**Serum + Tissue**	**-**	**-**	1,090	234	148	1,265	621	241	**431**
**Common variants in serum + tissue (% of tissue variants)**	**Serum + Tissue**	**-**	**-**	32.81	12.37	5.17	56.68	22.02	10.51	**17.195**

### Library preparation and exome sequencing

Due to the observed size distribution and the nucleosomal laddering, we sheared the cfDNA by ultrasonification in order to increase the amount of appropriately sized input molecules for library preparation. Most commercially available technologies for WES require amounts of < 1 μg genomic DNA as starting material. However, since the DNA yields from 200μl serum or plasma are typically in the low ng range, we aimed to perform WES from serum DNA by combining an ultra-low input library preparation protocol with a hybridization-based approach for exome enrichment. Starting from 10 ng of sonicated serum DNA, we generated indexed sequencing libraries from the six NSCLC and two control samples. Quality assessment confirmed sufficient yields above 200 ng as well as good qualities of the sequencing libraries with median sizes of 297 bp (Fig 1B).

Hybridization-based exome enrichment was performed using the Agilent SureSelect^*XT2*^ All Exon v5 + UTR target enrichment system. Compatibility of the SureSelect technology with ThruPLEX low input libraries has been shown in a previous study [41]. Here we combined the ThruPLEX-FD library preparation with the SureSelect technology for WES analysis of cfDNA. In each analyzed multiplex, we pooled equal amounts of cfDNA and corresponding tumor tissue libraries from three NSCLC patients as well as one library generated from control serum DNA. To further increase the complexity, captured libraries of each pool were split, amplified independently in two separate PCR reactions, and pooled again after amplification. To assess the quality of the enriched products, we performed fragment analysis. Consistent with the average size of the input libraries, both multiplexes revealed fragment sizes of approximately 295 bp and were sequenced on two lanes on the Illumina HiSeq instrument.

### Evaluation of cfDNA sequencing performance

In median, 161 million paired reads (range: 107–190 million) were obtained from serum DNA and approximately 145 million paired reads (range: 90–192 million) from the corresponding NSCLC tissues. We first examined the overall performance of our exome sequencing approach and data quality of serum DNA reads by calculating different quality metrics, including read count, library insert size, GC content, properly paired reads, enrichment efficiency, target coverage, and read count after post-processing (Table 2). We observed no difference in the alignment of serum and tissue reads: A mean of 86% reads from serum and 85% reads from tissue samples were uniquely aligned to the human reference genome (hg19), resulting in 130 million and 122 million perfectly mapped reads. After removal of PCR duplicates, we investigated whether the DNA shearing had a negative influence on the fragmented cfDNA molecules, which were already of mononucleosomal size before sonification. Estimation of the actual library insert sizes from patient sera using Picard revealed a median insert size of 166 bp, which is consistent with the median size of cfDNA fragments of 166 bp.

Regions with high or low GC content negatively affect library PCR amplification [42, 43] and target hybridization efficiency [44]. Thus, GC- or AT-rich regions might be underrepresented especially in cfDNA reads with an increased number of amplification cycles. Analysis of the GC composition revealed no differences between the GC contents of serum (mean 47%) and tissue samples (mean 46%), indicating that the target regions are equally represented in both specimen types.

**Fig 2:**
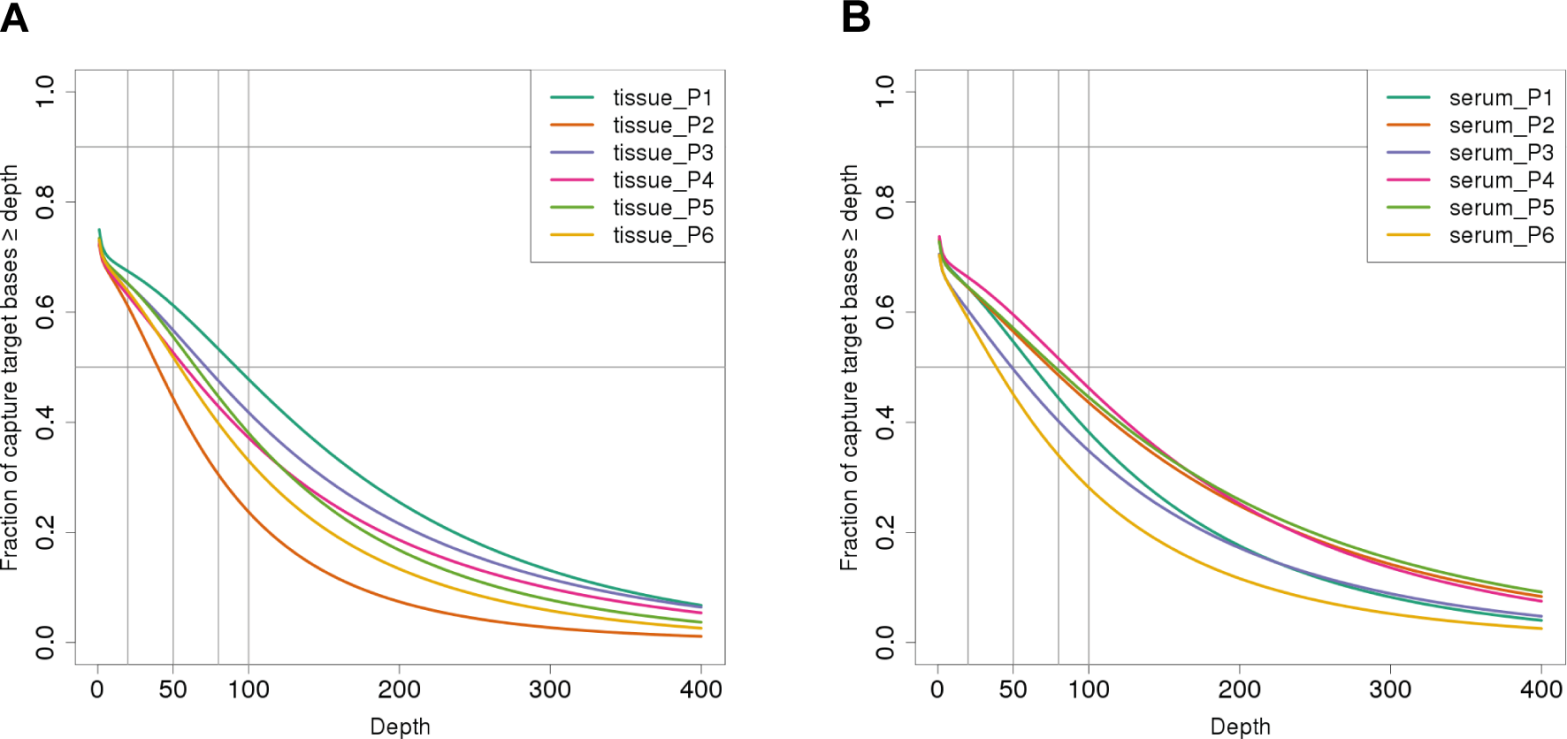
Target coverage distributions. Exome sequence coverages in primary NSCLC tissues (A) and cfDNA from corresponding serum samples (B).

Capture efficiency is a central aspect of hybridization-based exome sequencing. In order to evaluate the exome enrichment performance, we estimated the percentage of reads aligned to the target as well as the target region coverage using the R package TEQC [29]. A fraction of 84% of the uniquely and properly paired cfDNA reads were mapped to the target region, resulting in a median exome sequencing depth of 68.5x (range 38x to 85x, Fig 2A). No differences of on-target ratios between serum and tissue DNA were observed. About 64% of the target regions in serum (58–66%) and tumor tissue (61–67%) were sequenced with > 20x coverage (Table 2). Corresponding tissue exomes were sequenced with a median depth of 61x (range 39x to 92x, Fig 2B). Although on average fewer reads were obtained from tissue samples by equal uniquely mapped read fraction on target, the higher coverage of tissue samples might be a result of the increased number of duplicates in serum libraries due to the lower starting amount. A higher library complexity might also influence hybridization efficiency leading to the higher coverage.

In order to achieve only high confidence unique target reads for variant analysis, we performed stringent post-mapping read processing using RealignerTargetCreator and IndelRealigner from GATK. Mapped reads were locally realigned around known insertion and deletion sites from the 1000 Genomes Project [30] in order to reduce the number of mismatching bases, which are easily mistaken as SNPs. Furthermore, all Phred scores were recalibrated to more accurately represent the real error probability, taking into account known SNPs and specific positions on the reads. After post-processing, we retained a median of 15 million (range 11.1–26.8 million) de-duplicated high quality reads localized to the target regions from serum DNA and 41 million reads (range: 36.2–54.8 million) from tissue DNA (Table 2).

### Identification of high-confidence variants in serum and tissue

The main aim of this study was to compare variants from tumor tissues with those found in corresponding serum samples, independent of their somatic origin. Therefore, we assessed the common variants in serum and tissue pairs in order to examine the informative value of cfDNA and to which extent it represents the tumors´ genetic profiles. First, we called variants in the filtered reads of cfDNA and corresponding NSCLC exomes using the GATK HaplotypeCaller. Consistent with previous reports on WES without matched normal tissue [45–47], we identified mean numbers of 48,069 variants in tissue and 38,959 variants in serum samples. On average, 75% of the variants found in tissues and 78% of the variants found in serum samples were annotated as single nucleotide polymorphisms (SNPs) in the dbSNP (v129) database and therefore excluded from further analysis.

Next, we applied filters to remove low quality and germline variants for the serum vs. tissue comparison. We retained tissue variants with a mutant allele frequency between 20% and 80%, a minimum coverage of 20x, and a base quality ≥ 50, common in maximum two samples. Variants with an allele frequency above 80% were considered as homozygous germline variants and thus excluded from tissue as well as serum calls. As allele frequencies below 1% have been reported for somatic alterations in cfDNA [15], no lower frequency limit for calls in serum samples was used. Only variants with a sequencing depth < 10x were removed. These filtering steps led to a final data set of 2,557 (range: 1,892–3,322) high-confidence variants in NSCLC tissues and 1,416 (range: 589–4,105) in the corresponding serum samples (Table 2).

### Variants in cancer-associated genes

**Fig 3:**
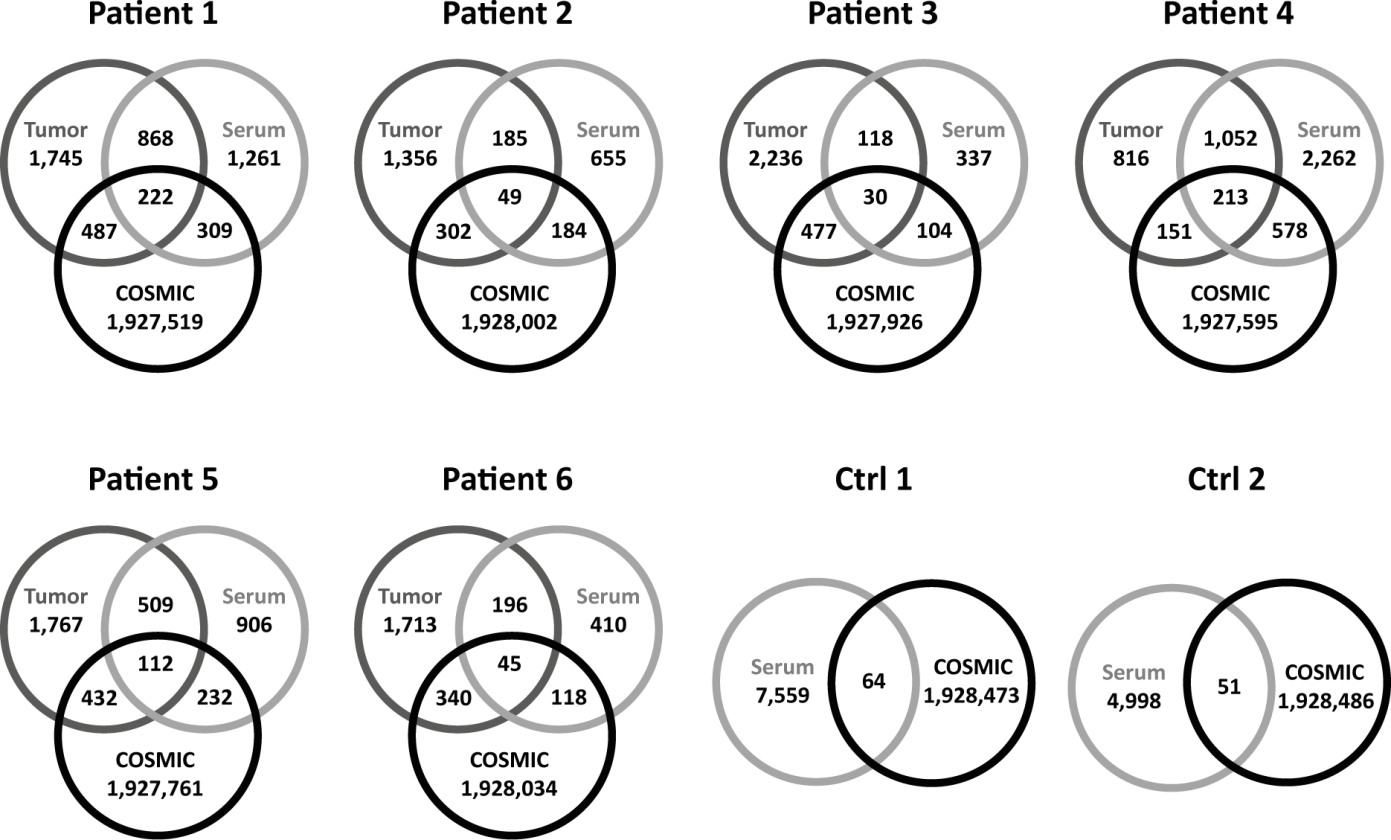
Comparison of shared and exclusive variants in serum and tumor tissue pairs compared to the COSMIC database of annotated somatic mutations.

To investigate to what extent cfDNA informs about cancerous molecular alterations, we compared the variants from tumor tissues with those found in the corresponding serum samples. Of the 2,557 high-confident tissue and 1,416 serum variants, a median of 431, representing 17.2% (range 5.2% - 56.7%; 148–1.265) variants were called in both specimen types (Fig 3, Table 2). We further analyzed the variants commonly identified in serum and matched NSCLC tissue from each patient. Consistent with previous findings [46, 47], we detected 39% (1,242) synonymous and 61% (1,966) non-synonymous variants, including a median of 238 (range: 76–654) missense variants among the coding alterations identified in the 6 patients. To identify cancerous somatic mutations in the absence of germline controls, we used the COSMIC database and the sets of NSCLC associated genes (S1 and S2 Tables). In the first approach, we compared serum and matched tissue variants with the COSMIC database of known mutation sites in human cancers (Fig 3). In the common variants of tissue and matched serum pairs, we identified 81 (range: 30–222) variants at COSMIC-annotated sites in each of the six patients. A median of 1.218 (range: 441–2.840) variants was identified in cfDNA, but not in the matched tissues, 1.970 (range: 967–2.713) were exclusive for the tumor tissues. Of these, an average of 254 serum and 363 tissue variants were found at COSMIC annotated sites.

In order to validate the performance of our approach, we performed WES of two serum pools (ctrl1 and ctrl2) from control subjects without evidence of NSCLC. Sequencing data were processed and variants filtered with the presented bioinformatical pipeline. Variant calling revealed 53,728 and 43,232 variants in cfDNA from ctrl1 and ctrl2. Upon SNP removal, a total of 11,449 and 8,966 variants were filtered using identical criteria as for the NSCLC serum variants. Filtration resulted in 7,623 and 5,056 remaining variant calls in cfDNA from pool ctrl1 and ctrl2, respectively. Thereof, only 64 and 51 variants were found at COSMIC annotated sites, including only 22 and 23 missense as well as 14 and 4 frameshift variants in ctrl1 and ctrl2, respectively (Table 2). Thus, the rate of coding COSMIC annotated mutations in pooled control samples is lower compared to NSCLC patient sera.

**Fig 4:**
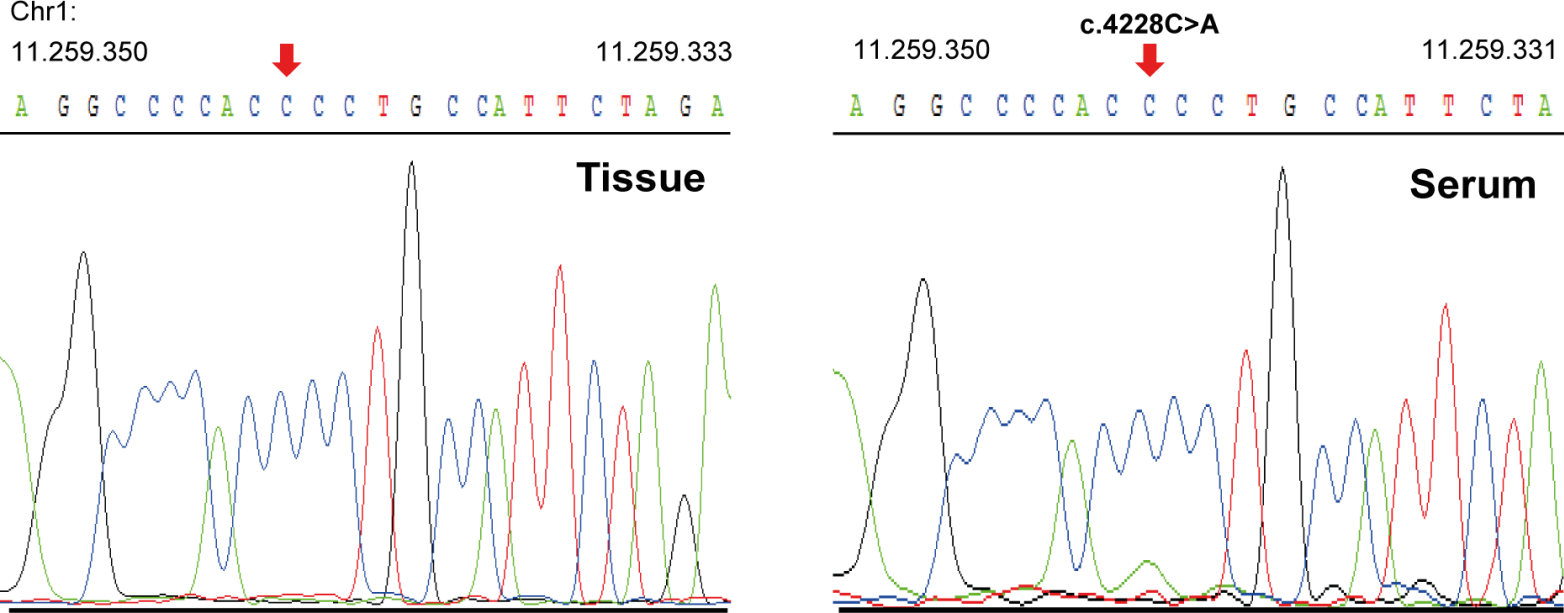
Sanger sequencing results from patient 4. Confirmation of the presence of the *MTOR* mutation c.4228C>A (p.P1410T) at a lower allele frequency in cfDNA and its absence in the corresponding primary tumor tissue.

We further used sets of genes, which had previously been found to harbor mutations associated with NSCLC to identify potential driver and prominent lung cancer mutations. Based on the TCGA and COSMIC databases as well as a published NSCLC panel [12], we designed sets of 58 and 45 genes for LUAD and SCC, respectively. By comparing thes target genes and the COSMIC reference to the WES data, we identified a broad range of NSCLC-associated somatic mutations in tissue and matched cfDNA (Table 3). Among the tumor tissue and cfDNA pairs, we identified COSMIC listed mutations in various kinases, including *PIK3CA*, *ALK*, *MAP2K3*, and *PAK2*. We further detected a splice site variant in the tumor suppressor gene *PTEN*. Moreover, cfDNA confirmed variants in *LRP1B*, *MET*, and the epigenetic modulator *KMT2C*. Apart from confirming variants detected in tumor tissues, cfDNA revealed additional variants of clinical relevance. For example, cfDNA of patient 4 revealed an *MTOR* mutation with an allele frequency of 15%, which was not found in tumor tissue. In order to support this finding, we performed Sanger sequencing of cfDNA and genomic DNA from the corresponding primary tumor tissue of patient 4. Sanger sequencing confirmed the presence of the *MTOR* mutation with a lower frequency compared to the wild type in serum cfDNA from patient 4. As expected, this mutation was not present in the tumor tissue (Fig 4). Although we identified common and COSMIC-annotated variants in serum and tissue pairs of all six patients, exome analysis of cfDNA could not confirm 2,126 (range: 967–2,716) mutations identified in primary tumor tissues. While none of the five *TP53* variants identified in tissues was found in the serum samples, cfDNA did not reflect the potential driver mutations in *PIK3CA* and *CDKN2A* from primary tissues of patients 1 and 2, respectively.

**Table 3:** Coding variants identified in tumor tissue and serum samples.

Case	Gene	Coding Consequence	Tissue	Serum
**P1**	***ALK***	p.E1419K, COSM159021		
	***VEGFB***	p.A194_A195dup		
	***PDGFRA***	p.S478P, COSM5008347		
	***MAP2K3***	p.L219W, COSM1579439		
	***ROS1***	p.R560H		
	***TP53***	p.R175H, COSM10648		
	***PIK3CA***	p.E545K, COSM763		
	***LRP1B***	p.D2670E		
**P2**	***NOTCH4***	p.L16_C17insL, COSM451257		
	***TP53***	p.Y236D, COSM43602		
	***CDKN2A***	p.R58*, stopgain, COSM12473		
	***FLT1***	c.1437-6dupT		
	***CSMD3***	p.S253C, COSM3644419		
**P3**	***VEGFA***	p.E273G		
	***KMT2C***	p.Tyr816fs, at COSM289942		
	***TGFA***	p.P54L		
	***NOTCH1***	p.P1210T		
	***FLT1***	p.R183L		
	***TP53***	p.P177L, COSM44097		
	***CSMD3***	splice site		
	***RYR2***	p.D2932H		
	***PRKCG***	p.M355I		
	***PRKCG***	p.V356F		
**P4**	***PTEN***	splice site		
	***PIK3CA***	p.I391M, COSM328028		
	***TP53***	p.K120_A129dup		
	***MTOR***	p.R32L		
	***MTOR***	p.P1410T		
**P5**	***MET***	p.T1010I, COSM707		
	***PAK2***	p.K128R, COSM4005518		
	***LRP1B***	p.G3615A		
	***EPHA3***	p.R914H, at COSM4002833		
**P6**	***TP53***	p.H179R, COSM10889		
	***PTCH1***	p.L420I		
	***ERBB3***	p.T906S		

## Discussion

Currently, cancer genome sequencing is used to identify genetic variants associated with malignant transformation. Since somatic alterations were first found in the blood of cancer patients, sequencing of cfDNA has been shown to be useful for minimal invasive diagnostics and therapy monitoring of malignant diseases [12, 48]. Few proof-of-concept studies have demonstrated the feasibility of WES of cfDNA for disease monitoring in several cancer entities, including NSCLC [11, 25, 26]. However, to which extent cfDNA represents the tumors´ molecular profiles in the circulation of cancer patients has not been systematically investigated yet. Moreover, standardized methods are needed to translate WES of cfDNA into clinical practice. Here, we present a robust experimental workflow for WES analysis of cfDNA and evaluate the molecular representation of the tumor exome in cfDNA by WES of matched tumor and serum samples from NSCLC patients.

Since most commonly available technologies for WES require large amounts of starting material which cannot be obtained from serum samples, DNA amount and complexity of the sequencing library are the limiting factors of hybridization-based WES, especially since more PCR cycles are required when the input material is limited. Based on previous reports on WES from low input samples and cfDNA, we used the ThruPLEX-FD Prep Kit (Rubicon Genomics) for library generation [11, 26]. Evaluation of cfDNA sequencing data illustrates the high performance of the established workflow, which combines the ThruPLEX-FD library preparation with the SureSelect technology for exome enrichment. We observed no differences in mapping performance, enrichment efficiency, target coverage, and sequencing depth between cfDNA reads compared to those from matched tissue samples.

We demonstrate the utility of WES for the identification of variants in serum samples from cancer patients. SNP removal and annotation of called variants using the COSMIC database of known mutations in cancer further showed that somatic mutations can be identified in the absence of germline controls. Our results from variant calling of matched serum and tissue pairs illustrate the informative value of cfDNA for cancer genotyping. While other groups used sequencing approaches primarily for limited numbers of prominent cancer associated genes [12, 48], we performed WES in order to estimate the representation of the tumor exomes in cfDNA. A median of 17.19% of the tissue variants (5.17% - 56.68%) was also found in the corresponding serum samples of the six tested NSCLC exomes. In addition, 81 (range: 30–222) of the common mutations in the serum and tissue pairs were at COSMIC-annotated mutation sites.

Although these data demonstrate the informative value of cfDNA at least for advanced cancers, the sequencing depth of WES represents a major limiting factor of the technology compared to targeted approaches, especially for the detection of low allele frequencies. While allele frequencies below 1% have been reported for tumor fragments in the circulation [15], the achieved sequencing depth eventually was too low to analyze the full representation of the tumor exome in cfDNA. Thus, an extensive fraction of variants, including *TP53* mutations in five patients, *PIK3CA* mutation in patient 1, and a *CDKN2A* mutation in patient 2, were exclusive for the primary tissues and not found in the corresponding sera, Their low abundances in the circulation could be influenced by several factors, including differences in tumor load, influences of therapy on the presence of cfDNA, or temporal variations of the abundance of cfDNA with respect to the tumor status.

Apart from shared mutations, 1.218 (range: 441–2.840) variants were identified in cfDNA only and were absent from the matched tissues. Although the cellular origin of these variants is difficult to trace, such variants may derive from different cell types and tissues in the body. Previous studies noted an accumulation of variants and mutations in different tissues within the same individual [49–52]. Thus, variants could originate from healthy cells, which accumulated mutations during differentiation and aging. However, analysis of cfDNA also allows for the identification of somatic mutations originating from metastatic lesions distinct from primary tumors [25]. Notably, we detected a *MTOR* mutation with a frequency of 15% in cfDNA of patient 4, which was not detected in primary tissue and confirmed this finding by Sanger sequencing. Such mutated alleles in cfDNA might have originated from an untested tissue lesion and thus provide complementary molecular information about therapeutically relevant mutations and the clonal heterogeneity of the disease.

In summary, we evaluated cfDNA to assess the exomes of six NSCLC patients in primary tumor and corresponding serum samples. We show that exome analysis of cfDNA is feasible for minimal-invasive characterization of tumor diseases. Our results provide evidence for cfDNA to inform about the molecular alteration in advanced cancer. Nevertheless, further evaluation and larger cohorts of different entities are needed to fully understand the value of WES of cfDNA as faithful representations of tumors.

## Supporting Information

S1 Fig Experimental and computational workflow for whole-exome sequencing of tumor tissues and cfDNA from corresponding serum samples. (TIF)

S1 Table LUAD panel of recurrently mutated genes in lung adenocarcinomas. (XLSX)

S2 Table SCC panel of recurrently mutated genes in lung squamous cell carcinomas. (XLSX)
