# Supplementary figures and images for: Low Input Whole-Exome Sequencing to Determine the Representation of the Tumor Exome in Circulating DNA of Non-Small Cell Lung Cancer Patients
Source: PLoS One. 2016 Aug 16;11(8):e0161012. doi: 10.1371/journal.pone.0161012 (PMC4987014; doi:10.1371/journal.pone.0161012)

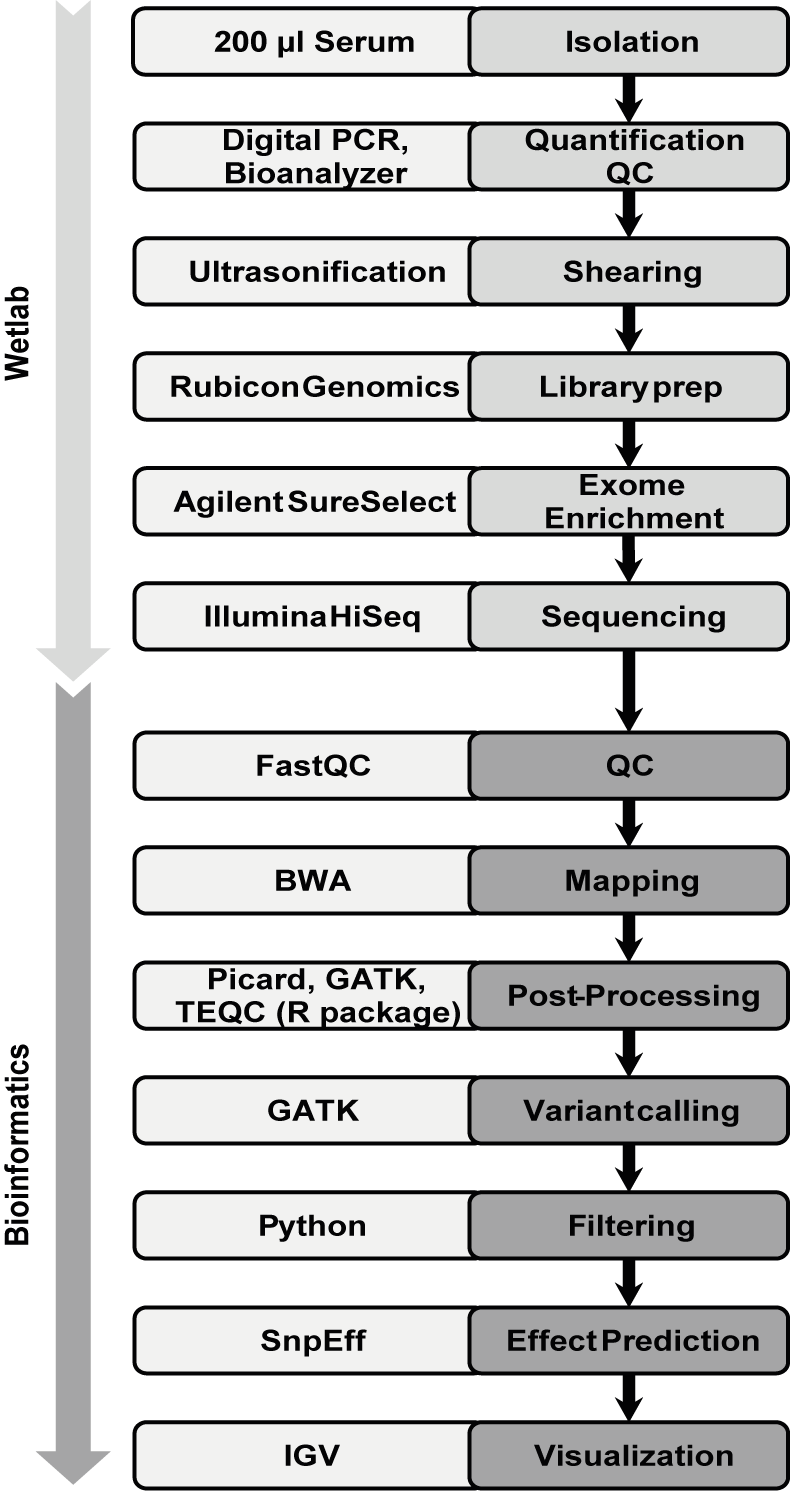

Supplement: S1 Fig — (TIF) [file pone.0161012.s001.tif]
